# Supplementary material for: Targeting Extracellular RNA Mitigates Hepatic Lipotoxicity and Liver Injury in NASH
Source: Cells. 2023 Jul 13;12(14):1845. doi: 10.3390/cells12141845 (PMC7614796; doi:10.3390/cells12141845)
Supplement: Supplementary file 1 [file cells-12-01845-s001.zip › cells-2303702-supplementary.pdf]

Supplementary Figures

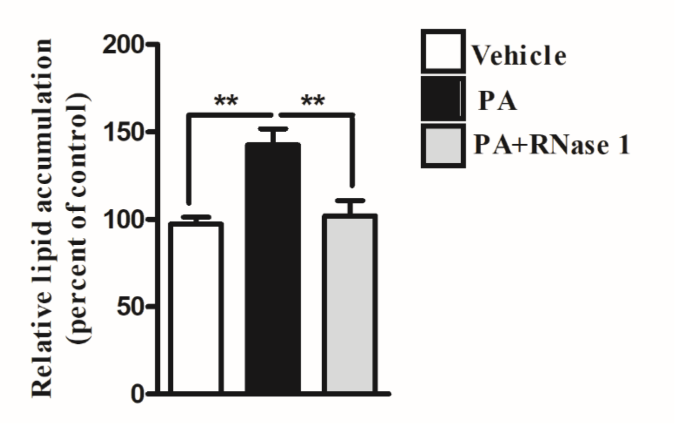

Supplementary Figure S1. eRNA release causes lipid accumulation in hepatocytes.
Oil Red O staining showing % lipid accumulation in hepatocytes in different experimental groups (Vehicle, PA, PA+RNase 1). Values are mean ± SEM, n=5, **p < 0.01, PA is compared to Vehicle and PA +RNase 1.

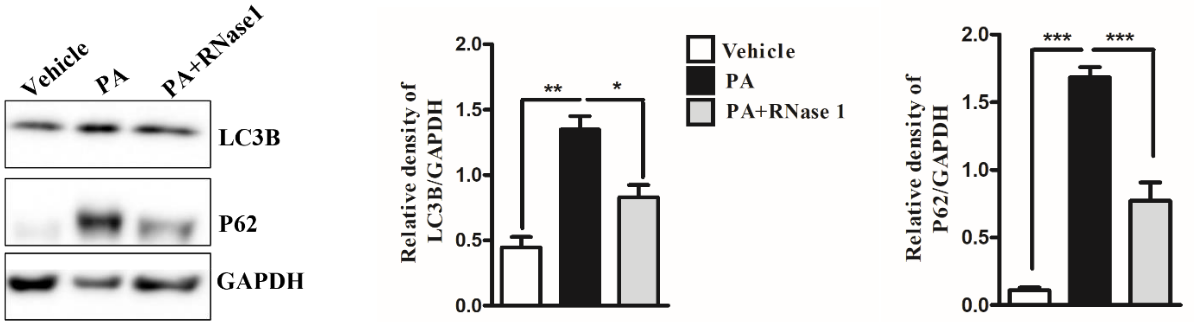

Supplementary Figure S2. RNase1 mitigates lipotoxicity induced autophagy block.
Representative immunoblots and densitometric analysis showing protein levels of LC3B and p62 in different experimental groups. Values are mean ± SEM; ***p < 0.001, n=5, **p< 0.01, compares Vehicle to PA and *p < 0.05 compares PA to PA+ RNase 1.

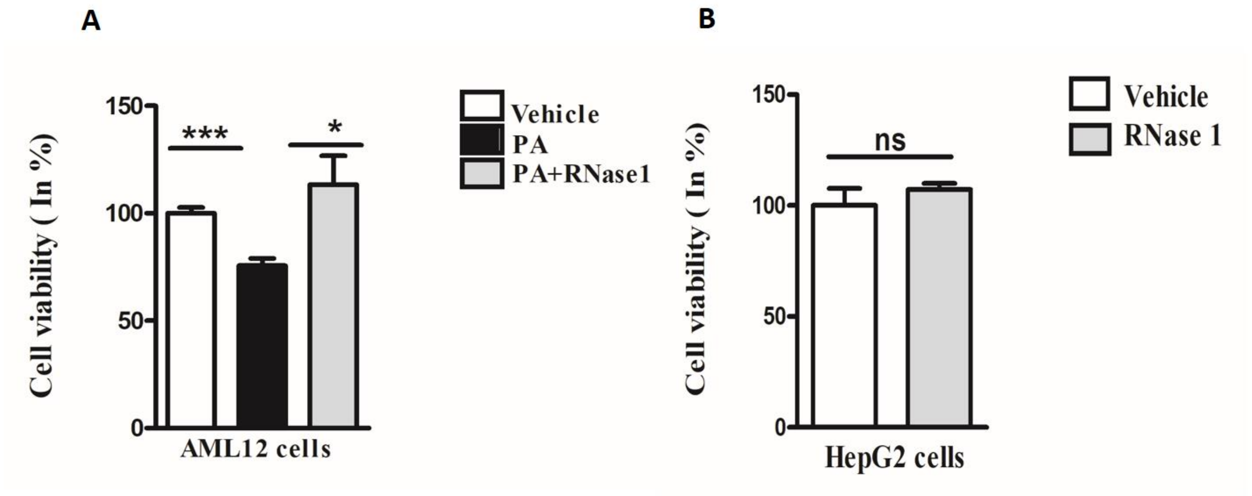

Supplementary Figure S3. Effect of RNase 1 on hepatic cell viability (A) % Cell viability shown through MTT assay among Vehicle, PA and PA+RNase1 treated AML12 cells. Data presented as mean ± SEM, n=5, ***p < 0.001 compares VC to PA and *p < 0.05 compares PA to PA+ RNase 1**. (B)** % Cell viability shown through MTT assay among Vehicle and RNase1 alone treated HepG2 cells. Data presented as mean ± SEM, n=5, ns indicates non-significant change between the two groups.

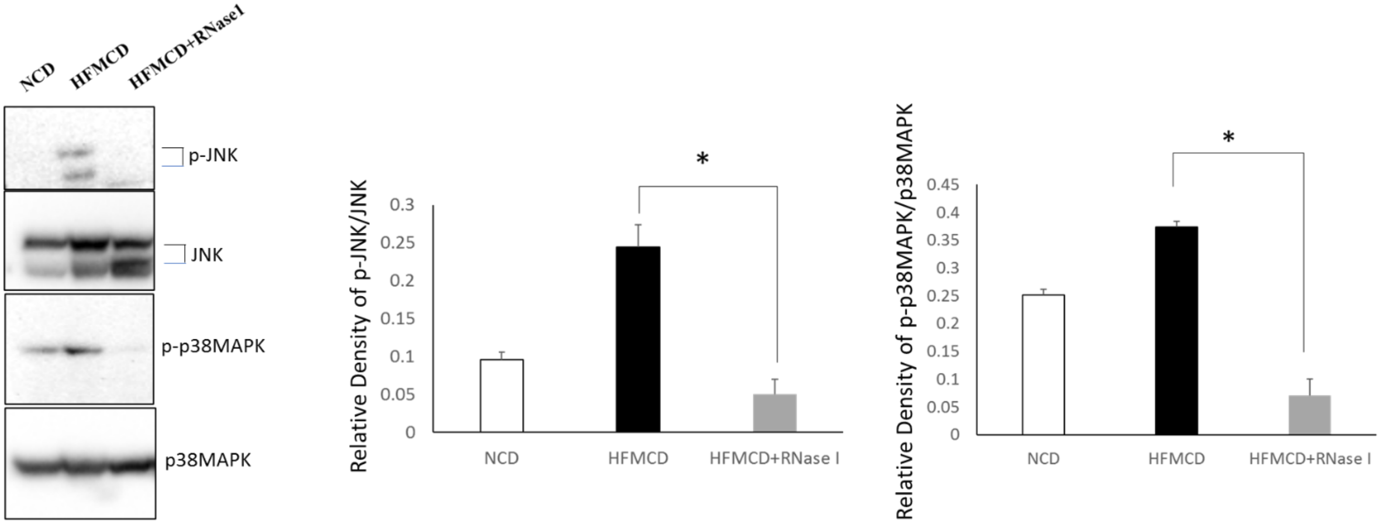

Supplementary Figure S4. Effect of RNase 1 on NASH induced stress in mouse liver
Representative immunoblots and densitometric analysis showing protein levels of p-JNK and p-p38MAPK in different experimental groups. Data presented as mean ± SEM, n=5, *p < 0.05 compares HFMCD to HFMCD+ RNase 1**.**

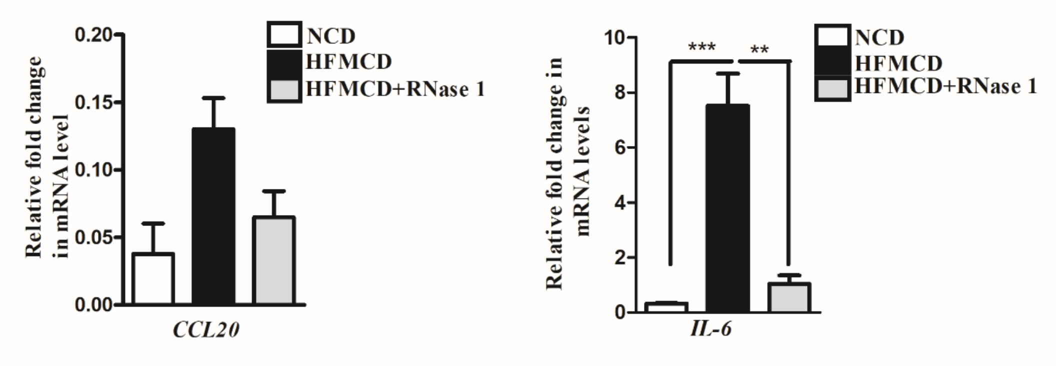

Supplementary Figure S5. RNase 1 administration reduces NASH induced liver inflammation. qRT-PCR analysis showing expression levels of CCL20 and IL6 in the livers of mice fed with NCD, HFMCD diet or HFMCD diet + RNase1.Values are mean ± SEM, n=5, **p < 0.01, HFMCD is compared to HFMCD+RNase1 and ***p<.001, NCD is compared to HFMCD.
